# RAGE Axis in the Pathogenesis and Treatment of CNS Neurodegeneration in Long-Term Hyperglycemia

**DOI:** 10.3390/ijms27041881

**Published:** 2026-02-15

**Authors:** Barbara Wasilewska, Urszula Mazur, Bernard Kordas, Patryk Mizia, Judyta Juranek

**Affiliations:** Department of Human Physiology and Pathophysiology, School of Medicine, University of Warmia and Mazury in Olsztyn, 11-041 Olsztyn, Poland; bachaw@uwm.edu.pl (B.W.); urszula.mazur@uwm.edu.pl (U.M.); bernard.kordas@uwm.edu.pl (B.K.); patryk.mizia@uwm.edu.pl (P.M.)

**Keywords:** hyperglycemia, diabetes, central nervous system, receptor for advanced glycation end-products, neurodegeneration, pathogenesis, treatment

## Abstract

Diabetes mellitus is one of the fastest-growing non-communicable diseases worldwide. The increasing global prevalence of diabetes has been accompanied by a corresponding rise in the incidence of diabetic micro- and macrovascular complications and related dysfunctions in the central nervous system. Studies demonstrated that patients with diabetes are more susceptible to cognitive impairment due to the diminished ability of neuronal cells to protect against increased production of reactive oxygen species and activated neuroinflammatory pathways. In the spinal cord, long-term hyperglycemia leads to neuronal dysfunction due to increased activation of glial cells and neuroinflammation and elevated oxidative stress, triggering micro- and macrovascular changes and leading to the development of peripheral nerve dysfunctions and neuropathies. Despite extensive efforts, however, the precise molecular mechanisms underlying the pathogenesis of diabetic complications have yet to be fully uncovered, proving to be a major hurdle in designing therapies to stop the progress of diabetes-triggered susceptible tissue and organ deterioration in affected subjects. In this review, we discuss in detail the role of the receptor for advanced glycation end-products (RAGE) and its major signaling partners in the development of CNS neurodegenerative changes in diabetes and the potential for novel biomarkers and treatments using targeting RAGE signaling axis.

## 1. Introduction

Diabetes mellitus is one of the fastest-growing non-communicable diseases worldwide. According to the International Diabetes Federation (IDF), one in ten adults globally live with diabetes [1]. The increasing global prevalence of diabetes has been accompanied by a corresponding rise in the incidence of diabetic micro- and macrovascular complications and related dysfunctions in the central nervous system. Evidence shows that long-term patients with diabetes are at an increased risk of developing dementia as in Alzheimer’s disease (AD) and motor disability as in Parkison’s disease [2]. And while the risk is lower as compared to other diabetic complications, with growing numbers of people living with diabetes, central nervous system (CNS)-related complications will be on the rise as well [3].

Glucose is essential for proper neuron function and maintenance, and its dysregulation has a profound effect on brain functionality [4]. Long-term hyperglycemia affects multiple tissues and organs, triggering pathological changes and leading to their dysfunction. Neurological disorders such as peripheral sensorimotor neuropathy, retinopathy or optic nerve ischemic neuropathy are among the most common complications of diabetes, and it is estimated that more than half of all long-term patients with diabetes will be affected by some form of neurological defects triggered by diabetes [3,5,6]. And while these complications have been well documented, structural and functional changes in central nervous tissue affected by long-term hyperglycemia are less studied and often remain overlooked. Evidence shows that both type 1 and type 2 diabetes lead to brain atrophy, observed reduction in white and gray matter and hippocampal injury [7,8]. Studies have demonstrated that patients with diabetes are more susceptible to cognitive impairment due to diminished neuronal cell protection against increased reactive oxygen species production and activated neuroinflammatory pathways. In the spinal cord, long-term hyperglycemia leads to neuronal dysfunction due to increased glial-cell activation, neuroinflammation, and elevated oxidative stress, triggering micro- and macrovascular changes, leading to the development of peripheral nerve dysfunction and neuropathies [9,10].

Multiple theories by which long-term hyperglycemia exacerbates diabetic complications exist; the most prevailing concept nowadays is that not only does hyperglycemia affect the permeability and structure of microvessels, but it also affects its neuronal component manifested by microstructural changes in affected tissues and organs alongside alternation in their vasculature, leading to their progressive dysfunction and, if not treated, irreversible pathological changes and organ failure [11]. Evidence indicates that the pathological changes underlying the development of diabetic complications might result from several interconnected molecular events and biochemical signaling cascades, such as excessive protein glycation, increased oxidative stress, local inflammation, and, last but not least, an upregulation of molecules involved in angiogenesis and cytoskeleton modification [12]. Despite extensive efforts, however, the precise molecular mechanisms underlying the pathogenesis of diabetic complications have yet to be fully uncovered, proving to be a significant hurdle in designing therapies to stop the progress of diabetes-triggered susceptible tissue and organ deterioration in affected subjects. 

**Figure 1:**
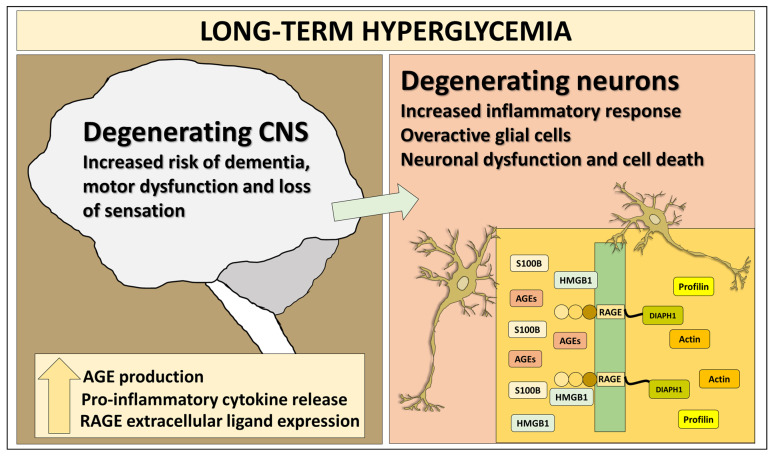
RAGE axis plays a significant role in many biochemical pathways leading to increased oxidative stress, protein glycation, cytoskeleton dysfunction and inflammation and contributing to the pathogenesis of neurological and neurodegenerative complications of diabetes. Both extracellular (AGEs, S100B and HMGB1) as well as intracellular (Diaph1 and related proteins, Profilin and Actin) contribute to the pathogenesis of neurodegenerative changes in diabetes, making it a difficult target for therapeutic interventions. Inset on the left depicts interaction patterns outside and inside the plasma membrane with HMGB1 and S100B acting as pro-inflammatory cytokines outside the cell, disturbing cellular homeostasis and leading to cell dysfunction.

In this review, we discuss in detail the role of the receptor for advanced glycation end-products (RAGE) and its major signaling partners in the development of CNS neurodegenerative changes in long-term hyperglycemia accompanying type 2 diabetes and summarize treatments using targeting directly and indirectly RAGE signaling axis (Figure 1).

## 2. RAGE Signaling Axis in the Pathogenesis of CNS Neurodegeneration

### 2.1. RAGE and AGEs

RAGE was first identified as a cell-surface receptor for Advanced Glycation End-products (AGEs) [13,14], the products of nonenzymatic glycation and oxidation of proteins/lipids that accumulate during physiological aging, but also in diabetes, inflammatory, and neurodegenerative diseases. It is a signal transduction receptor, and its activation triggers an increase in proinflammatory molecules, oxidative stressors and cytokines [15]. AGEs are constantly produced by the body and accumulate in greater amounts in older adults and patients with diabetes. By increasing oxidative stress and inflammatory processes, AGEs damage organs, including the brain [16]. In brain tissue, they are present in neurofibrillary tangles and senile plaques in patients with AD [17]. AGEs penetrate brain cells and activate the RAGE receptor, leading to increased expression of pro-inflammatory cytokines, adhesion molecules and the RAGE receptor itself, resulting in chronic inflammation, increased oxidative stress and tissue damage [17] ultimately contributing to cerebral vascular pathology [18].

The exposure of cerebral microvessels to hyperglycemic conditions or AGEs ex vivo resulted in significant disturbances in the distribution of tight junction proteins in the cell membrane, which determines the integrity of the brain–blood barrier (BBB). Studies have shown that treatment with glyoxal (GO) led to a significant reduction in the expression of claudin-5 and occludin within tight junctions (by approx. 20%). Similarly, the presence of methylglyoxal (MGO) caused a significant, approximately 20% reduction in tight junction staining for claudin-5 [19]. Furthermore, exposure of human brain microvascular endothelial cells (BMVECs) to high levels of glucose and AGEs led to a significant increase in ICAM and VCAM expression, enhanced leukocyte adhesion and migration to BMVEC monolayers, and increased brain–blood barrier (BBB) permeability in vitro [19]. It is reasonable to conclude that hyperglycemia and AGEs affect the reorganization of the actin cytoskeleton and the tightness of BBB. Patients with diabetes often have increased AGE deposition and increased expression of their RAGE receptor in the brain, which may mediate a common pro-inflammatory pathway leading to the development of neurodegenerative diseases. There is evidence that AGEs are overexpressed in the spinal cord in neurodegenerative diseases such as incurable and experimental forms of amyotrophic lateral sclerosis (ALS) [20,21].

A strong association between diabetes and cognitive impairment has been observed in AD, which is sometimes referred to as ‘type 3 diabetes’ [22,23]. One of the main pathologies identified in AD is the presence of excess β-amyloid, for which RAGE is also a receptor. RAGE expression occurs near sites of β-amyloid deposition [24], which interacts with the vascular system, affecting cerebral blood flow. This mechanism amplifies the cellular stress underlying neuronal dysfunction and dementia. Systemic infusion of β-amyloid and studies in genetically modified mice show that the interaction of β-amyloid with vascular wall cells expressing RAGE receptor leads to the transport of β-amyloid across BBB and to the expression of pro-inflammatory cytokines and endothelin-1. Inhibition of RAGE–ligand interaction reduces β-amyloid accumulation in the brain in a transgenic mouse model. These results suggest that vascular RAGE is a therapeutic target for inhibiting the pathogenic consequences of β-amyloid interaction with the vascular system, including the development of cerebral amyloidosis [25]. The importance of AGEs and RAGE in the pathogenesis of AD in patients with diabetes is also confirmed by immunohistochemical studies of human post mortem material. It has been shown that the brains of patients with concomitant AD and diabetes were characterized by higher levels of AGEs, a greater number of dense β-amyloid plaques, a higher number of RAGE- and tau-positive cells, and increased microglial activation compared to those of AD patients without diabetes. These results suggest that AGEs may promote a vicious cycle of oxidative stress, which may explain the more severe course and faster progression of the disease in patients with both diabetes and AD [26]. Furthermore, it has been shown that N-epsilon-carboxymethyl lysine (CML) staining intensity was increased in cortical neurons and blood vessels in post mortem material and correlated with the severity of cognitive impairment in elderly individuals with a history of diabetes and cerebrovascular disease, with minimal pathology characteristic of AD. This suggests that the accumulation of AGEs may contribute significantly to the development of vascular dementia [27]. Recent studies also demonstrated that there is a synergy between RAGE-AGE and β-amyloid binding, allowing for their enhanced penetration of BBB, increasing local cerebral inflammation and ROS release [28], further highlighting the role of RAGE signaling in the AD pathology. However, it must be noted that clinical and histopathological evidence shows that native formation of AGEs in the brain is not independently associated with AD-like dementia, rather it is one of the factors affecting brain function in physiological aging and the development of AD [29].

Studies in mice have shown that RAGE is present in both the gray and white matter of the brain. Higher RAGE expression was observed in the brains of people with diabetes in the internal capsule, the CA3 area of the hippocampus, the corpus callosum, visual cortex, ventral–posterior–lateral nucleus of the thalamus, cerebellum, substantia nigra, septal nuclei, medial prefrontal area, pons, brainstem and cerebellum. Higher RAGE expression in the diabetic brain was observed in hippocampal pyramidal neurons, dentate gyrus granule neurons, and glial cells: astrocytes, oligodendrocytes, and microglia. Compared to healthy individuals, a more than two-fold increase in total RAGE mRNA expression was found in the cerebral cortex and thalamus of patients with diabetes. It is suggested that RAGE signaling plays an important role in the pathogenesis of myelin damage in diabetes, and its absence partially protects the brain from its loss in several key areas of the brain, such as the internal capsule, corpus callosum, substantia nigra, thalamus, septal nuclei, the pons, and the cerebral peduncles. Interfering with AGE-RAGE signaling may provide therapeutic opportunities for patients with diabetes and neurological disease [30].

### 2.2. RAGE, HMGB1 and Toll-like Receptor 4 (TLR4)

HMGB1, home mobility group box-1, aka amphotherin, is a nuclear, non-histone DNA-binding protein characterized by high mobility within the cell nucleus [31]. In response to various stressors, HMGB1 is passively released from dying or damaged cells into the extracellular space [32]. The function of extracellular HMGB1 depends on the type of receptor it binds to, including receptors for advanced glycation end products (RAGE) and TLR4, which is the primary receptor for endogenous extracellular HMGB1 [31,33]. HMGB1 and TLR4 form a ligand–receptor pair involved in diseases characterized by cell death and brain-tissue damage [34], including diabetes [35] and AD [36]. Extracellular HMGB1 stimulates glutamate release, which mediates neuronal cytotoxicity [31]. Overstimulated neurons can actively release HMGB1, and cell apoptosis also increases HMGB1 levels, promoting further HMGB1 production [29]. Hyperglycemia increases the expression of RAGE, TLR4, and HMGB1 proteins, inducing the death of brain endothelial cells, astrocytes, and pericytes that form the BBB, as well as reducing its integrity through the degradation of tight junction proteins such as zonulin and occludens-1 (ZO-1) [37]. Increased BBB permeability leads to leakage from cerebral microvessels, resulting in memory loss [38]. It has been reported that BBB permeability is increased in patients with diabetes and dementia, and that this increase is associated with Aβ accumulation and apolipoprotein E (APOE) genotype in the brain [39]. The literature reports that the HMGB1–TLR4 signaling pathway may inhibit autophagy and promote apoptosis in structures involved in cognition. Activity of the HMGB1/TLR4 signaling pathway was observed in the hippocampus of diabetic mice mainly in the group with simultaneous diabetes and hypoxia. Transfection with siRNA targeting HMGB resulted in a significant reduction in HMGB1 and TLR4 expression, while increasing the levels of autophagy-related proteins: light chain 3 and Beclin 1. In the group of mice with diabetes and intermittent hypoxia, a reduction in the expression of activated caspase-3, an indicator of apoptosis, was also observed. In turn, direct binding of HMGB1 using specific anti-HMGB1 antibodies may prevent HMGB1-dependent pathogenesis by inhibiting the HMGB1/TLR4 signaling pathway in hippocampal cells. As a result, apoptosis is reduced while autophagy, which is protective for cells, is intensified [40].

Another pathway, HMGB1/RAGE/NF-κB, may also play a key role in the pathogenesis of hippocampal neuroinflammation in diabetic mice. Activation of this pathway leads to microglia activation and increased neuroinflammation. Under hyperglycemic conditions, the hippocampus shows increased HMGB1 expression at both the mRNA and protein levels. Overexpression of brain-derived neurotrophic factor (BDNF) inhibits excessive activation of the HMGB1/RAGE/NF-κB signaling pathway. It reduces HMGB1 expression, suggesting that BDNF may exert anti-inflammatory effects by modulating this signaling pathway and protecting hippocampal synapses in diabetic mice [41]. Increased levels of RAGE receptor expression and BBB vascular abnormalities are common features of diabetes and dementia [38,42]. Under hyperglycemic conditions, increased HMGB1 expression exacerbates vascular inflammation and endothelial cell dysfunction through activation of the NLRP3 inflammasome [43,44]. Disruption of the BBB is a cause of dementia in diabetes and AD (AD) [39,45], and one of the possible causes of this process may be pyroptosis inflammation-dependent cell death. The induction of pyroptosis is associated with the activation of the NLRP3 inflammasome, which responds to damage-associated molecular patterns (DAMPs) [46]. HMGB1, acting as a DAMP, exacerbates neuroinflammation in neurodegenerative diseases [47] and may initiate NLRP3 inflammasome activation [48]. Rats with type 1 diabetes subjected to middle cerebral artery occlusion showed elevated serum HMGB1 concentrations as well as increased expression of RAGE and TLR4 in the ischemic area of the brain. In contrast, treatment with niaspan, initiated 24 h after middle cerebral artery occlusion in diabetic rats, significantly reduced serum HMGB1 levels and attenuated RAGE and TLR4 expression in the ischemic brain compared to the control group [49,50].

An increase in extracellular HMGB1 was also observed in cerebrospinal fluid during the early stages of ischemic stroke, and this increase was more pronounced in animals with hyperglycemia [50]. In a mouse model of type 2 diabetes, increased HMGB1 protein was observed in the dorsal horn of the spinal cord, suggesting its role in mechanical allodynia. Elevated HMGB1 levels lead to astrocyte activation, which likely increases gene transcription and consequently modulates the synthesis of inflammatory mediators such as cytokines and chemokines IL-1, TNF-alpha, monocyte chemotactic protein, IL-6, which positively contribute to pain sensitization [51]. Furthermore, HMGB1 involvement in the induction of inflammation is signaled by the fact that after blocking HMGB1 with a specific antibody, cytokine levels in the spinal cord of diabetic mice were significantly reduced [52]. During diabetes, the expression of the RAGE receptor also increases [53,54], which, alongside TLR2 and TLR4, may mediate the pronociceptive effect of HMGB1 [52].

### 2.3. RAGE, S100B, S100A8

The S100 family of proteins, belonging to Ca^2+^ binding proteins, plays important roles in the regulation of basic cellular processes such as proliferation, differentiation and apoptosis. Low concentrations of S100 proteins may have neuroprotective effects, while elevated levels occur in pathological conditions and are often correlated with CNS damage, infection, ischemia or neurodegeneration [55]. The extracellular activity of S100 proteins is closely related to the multi-ligand RAGE receptor, whose expression and activity increase in conditions of hyperglycemia and type 2 diabetes [56,57]. The interaction of S100B with RAGE receptor leads to the activation of a signaling cascade involving MAPK kinases, including ERK1/2, p38 and JNK [58]. The S100B protein, acting through RAGE signaling pathway, may aid in the differentiation between hemorrhagic and ischemic stroke, as its concentration is significantly higher in the first hours after hemorrhagic stroke compared to ischemic stroke [59]. Specific astroglial abnormalities were also noted in this region of the brain, including reduced S100B content with simultaneous increased secretion and reduced glutamate uptake. These changes may be mediated by RAGE- and AGE-induced inflammation and may also impair glutamatergic neuronal communication in diabetic rats, contributing to cognitive deficits [56].

The addition of S100B significantly reduced glucose uptake in both astroglial cells and hippocampal slices, while incubation of these cells with anti-S100B antibody and blockade of RAGE receptor with anti-RAGE antibody restored glucose uptake to baseline levels. It has also been shown that the reduction in glucose uptake caused by S100B via RAGE receptor is dependent on the ERK1/2 pathway [60]. The study of the effects of glucose, MGO and CML on oxidative stress, metabolism and astrocyte function in the hippocampus, found that exposure to AGE precursors leads to a reduction in glutamate uptake and a decrease in S100B protein secretion [61]. On the other hand, it has also been shown that astrocytes cultured in high-glucose medium exhibit reduced S100B protein secretion but without an accompanying change in glutamate uptake [56]. Astrocytes and C6 glioblastoma cells cultured in high glucose conditions exhibited an atypical appearance and lower density, and their proliferation was reduced by approximately 25% in astrocytes and 47% in glioblastoma cells, respectively. High glucose reduced A100B secretion in astrocytes, while glioblastoma cells remained resistant to this effect, indicating clear differences in the signaling pathways regulating the secretion of this protein. Based on the neurotrophic effects of S100B protein in vitro, the data suggests that chronically elevated glucose levels affect glial activity by reducing extracellular secretion of S100B, which in turn may affect neuronal activity and survival. Such changes in astrocytes may contribute to the cognitive deficits and other impairments observed in patients with diabetes mellitus [62]. Diabetic animals show an increase in depressive and anxious behavior, associated with an increase in oxidative stress parameters and AGE and RAGE levels in the prefrontal cortex and hippocampus, as well as a decrease in S100B levels. Melatonin treatment has been shown to improve AGE, RAGE and S100B levels in both brain areas, while alleviating depressive and anxious behavior. These results emphasize that oxidative stress, AGE, RAGE and S100B may play an important role in the pathophysiology of depression, and inhibition of both the AGE/RAGE pathway and oxidative stress appears to be a possible mechanism by which melatonin exerts antidepressant and anxiolytic effects in diabetic rats [63]. Elevated plasma AGEs contribute to hyperalgesia through a tyrosine phosphatase 1B (PTP1B)-dependent mechanism involving the Src/NMDAR signaling pathway in the dorsal horn of the spinal cord. Targeting the AGEs–PTP1B–NMDAR pathway may provide new therapeutic strategies for diabetic neuropathic pain treatment(DNP) [64].

Not only the S100B protein, but also the S100A8 may be involved in metabolic disorders leading to neurodegeneration and the development of AD. Medium- and high glucose concentrations, mimicking prediabetes and diabetes, caused a statistically significant release of S100B and S100A8 proteins from dopaminergic neurons into the extracellular space. This phenomenon suggests that these proteins may have a neuroprotective effect, supporting neuron survival under conditions of disturbed metabolic homeostasis [65].

## 3. RAGE, Diaph1 and Profilins

### 3.1. Diaph1

Diaph1, protein diaphanous homolog 1, a member of diaphanous-related formins (DRFs) [66], is a cytoplasmic protein involved in the regulation of the cellular cytoskeleton in tandem with its cytoplasmic binding partner, Profilin1 (Pfn1). It is encoded by the DIAPH1 gene located on chromosome 5 [67] and has 14 splice variants. Mutations in the DIAPH1 gene cause autosomal dominant non-syndromic sensorineural deafness with or without thrombocytopenia [68,69]. Furthermore, the loss of DIAPH1 gene in humans leads to seizures, cortical blindness, and microcephaly syndrome (SCBMS) [70] with or without immune and mitochondrial dysfunctions [71]. Interestingly, a study from 2021 on PFN1 mutations in ALS revealed a relation between these mutations and the increased expression of DIAPH1 gene, affecting formin-driven actin polymerization [72]. Numerous evidence, detailed by our group elsewhere [73,74] shows that both RAGE and Diaph1 are involved in prolonged hyperglycemia-driven neurovascular, structural and metabolic changes in cells and tissues most susceptible to prolonged elevated blood glucose levels and related glucose toxicity.

### 3.2. Profilins

Profilins, encoded by four genes (PFN1–PFN4), exhibit distinct expression patterns in mammals and contain conserved actin-, phosphatidylinositol 4,5-bisphosphate (PIP2)-and poly-L-proline-binding domains [75,76,77,78]. In mammalian CNS, two profilin isoforms are expressed: profilin-1 (Pfn1) and profilin-2a (Pfn2a), with Pfn2a being the predominant neuronal isoform. Both isoforms show overlapping subcellular localization at pre- and postsynaptic sites and in the nucleus and undergo activity-dependent translocation into dendritic spines [79,80]. Despite these similarities, Pfn1 and Pfn2a serve distinct functions in synaptic actin regulation: Pfn1 is mainly required for dendritic spine formation, whereas Pfn2a supports spine stabilization, synaptic function, and activity-dependent structural plasticity. Consequently, partial functional redundancy exists, but loss of one isoform cannot be fully compensated by the other form [81,82,83].

Pfn1 is a small (12–15 kDa), multifunctional actin-binding protein that plays a pivotal role in cytoskeletal rearrangement and redistribution by promoting actin polymerization and filament remodeling [84]. In addition to facilitating the conversion of G-actin to filamentous (F) actin, Pfn1 interacts with a broad range of proline-rich proteins, including members of the mammalian enabled (Mena)/vasodilator-stimulated phosphoprotein (VASP) family [85]. In neurons and in other cell types, profilin is thought to participate in the reorganization of the actin cytoskeleton by binding to actin and to a plethora of cytoskeleton-regulating proteins with proline-rich domains, such as Ena/Mena/VASP family members, and N-WASP [86,87]. Together, profilins and Mena/VASP proteins are considered essential for submembranous actin filament formation and structural organization. Pfn1 activity is tightly regulated by phosphorylation and is a downstream target of Rho-associated kinase (ROCK) within the integrin-linked kinase signaling pathway. ROCK-mediated phosphorylation of Pfn1 at Ser137 functionally inactivates the protein [88], resulting in reduced radial lamellipodia formation in Schwann cells [89]. Pfn1 as a key effector of integrin-linked kinase/Rho/ROCK signaling and an essential regulator of Schwann cell morphology and peripheral nervous system development [89]. In the nervous system, Prf1 has been implicated in neurodegenerative disorders, including Huntington’s disease, and mutations in the PFN1 gene have been linked to familial amyotrophic lateral sclerosis [88,90]. More recently, PFN1 was also identified as a modulator of phagocytosis in a CRISPR screen performed in human-induced pluripotent stem cell-derived microglia-like cells [91]. Reduced expression of PFN1 and β-actin (ACTB) has been detected in the sciatic nerve following six months of type 1 diabetes [92]. Accumulating evidence has indicated the important role of Pfn1 in inflammatory and neurological diseases [93,94]. Pfn1 transcripts are more abundant in microglia than in neurons, suggesting a potential role in neuroinflammation [95,96,97,98,99]. Endothelial barrier damage further enhances neutrophil–endothelial interactions, amplifying inflammation during endotoxemic responses.

Pfn1 functions as a central integrator of cytoskeletal signaling [100]. Accordingly, profilins participate in distinct signaling pathways, including regulation of ADP–actin monomers via the Srv2/CAP pathway. Rho/Rac signaling mediated by Pfn2 [101] and the PI3K/AKT pathway associated with profilin 1 [102]. Furthermore, Pfn1 functions as a key mediator of AGE-induced oxidative stress, inflammation, and vascular dysfunction in diabetes. AGE stimulate Pfn1 expression, which in turn promotes ROS production and activation of the RhoA–ROCK1 signaling pathway, leading to endothelial cell injury as well as inflammatory responses by enhancing ICAM-1 expression [103,104]. Moreover, Pfn1 appears to participate in a feed-forward loop with AGE and RhoA–ROCK signaling, amplifying oxidative stress and inflammatory signaling in diabetic vascular disease [105].

Pfn2, the major splice variant encoded by the PFN2 gene, is predominantly expressed in neuronal cells and serves as a key regulator of neuronal growth, development, and dendritic spine formation. Unlike Pfn1, Pfn2 primarily inhibits actin polymerization by sequestering actin monomers. In contrast, the survival motor neuron (SMN) protein stabilizes filamentous actin under destabilizing conditions, such as urea exposure, thereby promoting actin polymerization [106]. In motoneurons, Pfn2 is highly enriched and colocalizes with SMN in the cytoplasm and nuclear gems through an interaction with the poly-L-proline (PLP) domain encoded by exon 5 of SMN1 [106,107,108,109] This interaction enhances F-actin formation and contributes to cytoskeletal stability [109]. The modulation of the neuronal actin cytoskeleton by free Pfn2 is mediated, at least in part, through its interaction with ROCK [106]. In neurons, ROCK directly associates with Pfn2, leading to increased actin filament stability and a concomitant inhibition of neurite outgrowth [101,107]. In addition to Pfn2, ROCK directly or indirectly phosphorylates other actin-regulatory proteins, including myosin light chain phosphatase (MLCP) and cofilin, both of which play critical roles in actin dynamics and neurite extension [108,109,110].

Alternative splicing of the PFN2 gene gives rise to two isoforms, profilin-2a and profilin-2b [111]. Among these, profilin-2a exhibits a higher affinity for proline-rich ligands and is considered the functionally dominant isoform in neurons. Profilin-2a has been shown to regulate neuritogenesis [99]. Notably, profilin 2a was the first actin-binding protein demonstrated to relocalize to postsynaptic sites in response to NMDA receptor activation and calcium influx—a signaling cascade essential for long-term potentiation [77]. Sharma et al. demonstrated that elevated PFN2 levels impair neurite outgrowth and axon pathfinding by disrupting the tightly regulated dynamics of the actin cytoskeleton in a spinal muscular atrophy (SMA) PC12 cell model [112]. Pfn2 is highly enriched in postsynaptic compartments [113,114], whereas actin is concentrated at both presynaptic terminals and postsynaptic densities [106]. Pfn2 directly interacts with the PLP domain of Piccolo, a presynaptic scaffold protein that regulates neurotransmitter release by promoting F-actin assembly [106,115]. PFN2 knockout mice display defects in synaptic actin polymerization and increased neurotransmitter release, indicating that Pfn2 plays a functional role in vesicle exocytosis [107,116]. Profilin was shown to be a necessary element in the following activity-dependent stabilization of dendritic spine morphology, which is irreversible for at least several hours, further underlining analogies to long-term electrophysiological plasticity [77,78]. Importantly, activity-induced profilin targeting to synapses has been demonstrated in vivo in the fear-conditioning paradigm of amygdala-dependent learning [106] as well as lengthening of postsynaptic densities, an important factor in synaptic plasticity (Figure 1).

## 4. RAGE Signaling Axis—Therapeutic Potential

Studies agree that hyperglycemia modulates RAGE pathway in the CNS. RAGE knockout mice, compared to wild-type mice, were protected from hippocampal-dependent spatial memory deficits in the streptozotocin-induced (STZ) diabetes model. Diabetic wild-type mice exhibited increased RAGE expression in neurons and glia of the hippocampus, while RAGE-deficient mice maintained normal cognitive performance despite severe hyperglycemia. FPS-ZM1, a RAGE agonist treatment, prevented spatial memory loss in these animals [117] indicating that RAGE signaling contributes to cognitive dysfunction under hyperglycemia, particularly affecting hippocampal synaptic plasticity and memory. This finding aligns with earlier observations in long-term diabetic mice (18–33 weeks), where an increase in RAGE is observed in both neuronal and glial cells. Current evidence strongly supports the mechanism by which diabetes-induced AGEs-RAGE interaction provokes neuroinflammation and neuronal injury in the CNS, contributing to cognitive decline. For example, pharmacological blockade of RAGE has been shown to have neuroprotective effects. In hyperglycemic mice, the small-molecule RAGE inhibitor FPS-ZM1 preserved memory performance and RAGE-neutralizing antibodies were reported to reduce diabetes-related synaptic and electrophysiological deficits in hippocampal circuits, thereby maintaining normal long-term potentiation [117]. These results reinforce earlier therapeutic observations with experimental RAGE antagonists (like Azeliragon/TTP488), which were designed to mitigate RAGE-mediated tissue damage. Although no RAGE inhibitor has yet reached FDA approval [118], the consistency of in vivo findings across genetic knockouts, antibodies, and small molecules underscores RAGE as a key driver of diabetic neurodegeneration and a viable therapeutic target [118]. Additionally, converging evidence implicates RAGE in diabetes-exacerbated Alzheimer’s pathology. RAGE on brain endothelium transports amyloid-β (Aβ) from circulation into the brain, while RAGE signaling in neurons can upregulate β-secretase (BACE1) [119]. Thus, diabetes may worsen amyloid accumulation via RAGE, providing a mechanistic link between diabetes and AD. Soluble RAGE (sRAGE), a decoy form of the receptor, is thought to sequester circulating AGEs/Aβ and reduce their binding to cell-surface RAGE [120]. The protective association of higher sRAGE with better cognitive outcomes in people with diabetes (discussed below) further validates the detrimental role of active RAGE signaling in the diabetic brain.

### 4.1. RAGE-Related Pathways in Diabetes-Driven CNS-Neurodegenation

Beyond classical NF-κB-mediated inflammation, RAGE-interacting pathways also drive neurodegeneration in diabetes. A direct interaction has been discovered between RAGE’s cytosolic tail and Receptor-Interacting Protein Kinase 1 (RIPK1), a kinase that regulates inflammation and cell death. The study showed that hyperglycemia induces RAGE-RIPK1 binding in microglia. That triggers RIPK1 phosphorylation and downstream neuroinflammatory signaling [118]. This RAGE-RIPK1 axis was found to impair synaptic transmission and memory. The use of a specific blocking peptide reduced microglial activation and significantly alleviated inflammation-linked cognitive deficits in diabetic mice. Mechanistically, the C-terminal cytosolic domain of RAGE (amino acids 362–367) binds to a motif in the RIPK1 death domain. The mutation of this site or the competitive inhibition of it with a peptide enabled researchers to prevent RIPK1-driven microglial overactivation [118]. This is a novel insight, as it identifies a precise protein–protein interaction through which RAGE transduces the effects of hyperglycemia inside microglia. It also provides a new therapeutic strategy. It allows targeting of RAGE’s intracellular tail (rather than its ligand-binding outside) to uncouple it from pro-degenerative signaling. Notably, patients with diabetes in this study had elevated plasma RIPK1 levels correlated with poorer cognition, reinforcing the clinical relevance of RAGE-RIPK1 mechanism [118]. This line of research expands our understanding of how RAGE functions not only as a receptor but also as a scaffold for signalosomes (such as the RIPK1 complex) that drive neuroinflammation and perhaps necroptosis in diabetic brains.

Another important pathway involves HMGB1, a RAGE ligand that is released during tissue stress. HMGB1 levels are elevated in diabetes [121] and can activate RAGE much like AGEs. Recent studies implicate HMGB1-RAGE interactions in diabetic neuroinflammatory responses. For instance, in models of diabetic neuropathic pain, HMGB1 from hyperglycemic tissues sensitizes neurons via RAGE. High glucose causes HMGB1 release, which binds RAGE on dorsal root ganglion neurons, increasing excitatory TRPV1 currents and pain behavior. Inhibiting HMGB1 or RAGE in those models prevented neuronal hyperexcitability and pain hypersensitivity [121]. While this pertains to peripheral nerves, it highlights a “sterile inflammation” mechanism likely at play in the CNS as well. HMGB1 from chronically stressed diabetic tissues (or activated glia) can engage RAGE on microglia and astrocytes, amplifying cytokine release. In fact, the HMGB1-RAGE axis may contribute to neuroglial activation in diabetic encephalopathy, like peripheral neuropathy. Concordantly, diabetic rodents show increased RAGE in spinal cord- and brain microglia, with RAGE signaling required for full activation of the NLRP3 inflammasome and cytokine maturation [118]. Novel evidence suggests RAGE signaling interfaces with inflammasome pathways. RAGE activation in hyperglycemic microglia was found to trigger NF-κB and prime NLRP3, facilitating IL-1β release and neuronal injury [118]. These emerging insights into RAGE’s crosstalk with RIPK1, HMGB1, and inflammasomes significantly broaden the understanding of its role beyond simple NF-κB activation, painting RAGE as a central orchestrator of multiple pro-degenerative signals in diabetes.

### 4.2. Human Studies

Clinical research in humans further supports RAGE axis as a mediator of diabetic CNS injury. A cross-sectional study of type 2 diabetes patients demonstrated that circulating markers of RAGE pathway are associated with cognitive impairment [120]. Patients with mild cognitive impairment (MCI) had significantly higher serum AGE levels and sRAGE levels compared to diabetic controls without MCI [120]. Each unit increase in serum AGE peptide level was associated with a 72% rise in odds of MCI, whereas each unit increase in sRAGE was protective, reducing MCI risk by ~54% [118]. Moreover, sRAGE levels inversely correlated with executive function (TMT-B test time) and AGE levels positively correlated with deficits across multiple cognitive domains. These clinical findings suggest that an overactive AGE-RAGE axis (high ligand, low decoy receptor) contributes to early cognitive decline in diabetes [120]. The authors concluded that RAGE pathway partially mediates AGE-induced MCI in patients with diabetes and proposed serum AGE and sRAGE as potential biomarkers or intervention targets for early diabetic cognitive decline [120]. A clinical study of skin biopsies from patients with diabetes and peripheral neuropathy found that RAGE expression was markedly upregulated in cutaneous nerve fibers and endothelial cells of patients with severe neuropathy [122]. Although this study focused on peripheral small fibers, similar microvascular and neuroinflammatory damage may occur in the central nervous system via RAGE. Post mortem analyses have noted increased RAGE and its ligands (CML AGEs, HMGB1, S100B) in the brains of diabetics and in AD patients with comorbid diabetes [123]. Chronic diabetes also correlates with higher brain levels of oxidative AGEs (MGO derivatives) that can engage RAGE [123]. Clinical biomarker studies show systemic inflammation in diabetes (high TNF-α, IL-6) correlates with cognitive decline, implicating RAGE-agonist cytokines as well [124]. Although human interventional data is limited, one trial reported that circulating sRAGE increased when patients were treated with statins and insulin sensitizers. It suggests that some standard therapies might indirectly modulate RAGE axis in humans [125]. Overall, human studies confirm that elements of RAGE pathway are measurable in patients and often correlate with the severity of diabetic neurological complications.

### 4.3. Large Animal Models

Insights from non-rodent models strengthen the link between RAGE signaling and diabetic neurodegeneration. Studies from diabetic pigs demonstrated a significant loss of small, myelinated fibers in the sciatic nerve along with markedly elevated immunoreactivity of RAGE and its ligand S100B in nerve tissue [123]. Highlighting the relevance of pig as a good translational model with a nervous system, size and complexity closer to that of humans. Non-human primate studies underscore the impact of diabetes on the brain, although direct measurements of RAGE are scarce. Accelerated brain aging and pathology have been documented in spontaneously diabetic cynomolgus monkeys, which develop cognitive deficits and AD-like changes, including more amyloid plaques at younger ages than normoglycemic monkeys [126]. Another study provided evidence that diabetes in middle-aged monkeys accelerated Aβ plaque deposition and increased GM1 amyloid levels (a seed for Aβ aggregation) in the brain [126]. Although RAGE was not explicitly measured, the authors speculated that diabetes-related inflammation and possibly glycation contribute to this accelerated pathology [126]. Given RAGE’s known role in mediating Aβ influx into the brain and microglial activation, it is plausible that RAGE axis contributes to these primate findings. Another long-term study in macaques, which lasted 3 years, showed that treating diabetic monkeys with metformin-preserved cognition and slowed brain aging [127]. Metformin is not a direct RAGE inhibitor, but it can reduce RAGE ligand levels and, consequently, the downstream damage. The fact that improving metabolic control and reducing oxidative stress in a primate model translated into measurable neuroprotection suggests that pathways such as the AGE-RAGE pathway may have been mitigated. Non-rodent models confirm that diabetic milieu leads to RAGE upregulation and neural injury. They also provide high-fidelity testing possibilities for RAGE-targeted interventions.

### 4.4. Direct RAGE Antagonists

Among many synthetic RAGE inhibitors and/or modulators commercially available for research and development, three, namely: Azeligaron (TTP488), FPS-ZM1 and sRAGE, have been most extensively tried and tested in pre-clinical and clinical trials. These substances either block (Azeliragon, FPS-ZM1) or decoy RAGE binding with extracellular ligands such as AGEs, HMGB1, S100s or Aβ, inhibiting RAGE downstream signaling pathways thus affecting cellular inflammatory and oxidative stress response in affected tissues (Table 1).

### 4.5. Indirect Modulation of RAGE

Several pharmacological agents developed to target other pathways have been found to modulate the RAGE axis or its ligands, thereby conferring neuroprotection in diabetic contexts.

#### 4.5.1. Metformin

Beyond improving glycemic control, metformin can reduce AGE/RAGE-mediated damage. In diabetic db/db mice, chronic metformin treatment significantly decreased RAGE expression in the brain vasculature and reduced Aβ influx across the BBB [139]. One study showed that 6 weeks of metformin (and other antidiabetics) in db/db mice lowered RAGE levels at the BBB and cut Aβ accumulation in the hippocampus, resulting in improved memory performance. By suppressing RAGE and related NF-κB activation at the BBB, metformin partially restored the balance of Aβ transport (reducing pathogenic influx) [139]. Clinically, this corresponds with data indicating that metformin users experience slower cognitive decline than non-users, potentially due to reduced RAGE ligand burden and inflammation. Another study reported that metformin slowed brain aging in male macaques and, mechanistically, was linked to lower markers of brain inflammation and metabolic stress [127], consistent with the dampened AGE-RAGE activity. Metformin thus exemplifies an agent that, while not a direct RAGE inhibitor, indirectly downregulates the RAGE pathway through glycemic control and AMPK-driven reduction in AGE formation [140].

#### 4.5.2. Thiazolidinediones

Pioglitazone, a PPAR-γ agonist used to treat type 2 diabetes, has shown cognitive benefits in diabetic models by modulating RAGE-related inflammation. In high-fat diet/STZ diabetic mice, pioglitazone treatment reversed learning and memory impairments and significantly lowered brain levels of RAGE, NF-κB, and BACE1, along with reducing Aβ_40/42_ deposition [141]. Treated mice showed improved maze performance, whereas untreated diabetic mice had elevated RAGE and cytokine levels in the hippocampus. The improvement in cognition correlated with pioglitazone’s effect in attenuating hyperglycemia and dyslipidemia, which lowers AGE production, as well as with a direct suppression of neuroinflammatory genes [141]. The fact that inhibiting NF-κB and RAGE expression was noted suggests PPAR-γ activation can transcriptionally repress components of the RAGE axis. These results echo other reports where pioglitazone or rosiglitazone reduced diabetes-induced IL-1β and TNF-α in the brain and improved memory, presumably by interrupting the AGE-RAGE-NF-κB positive feedback [142,143]. Although a human trial of pioglitazone in AD did not show significant benefits, in the specific context of diabetic encephalopathy, this drug appears to mitigate RAGE-associated damage. Its dual action, through peripheral metabolic improvement and central anti-inflammatory effect, makes it a valuable example of therapy repurposing.

#### 4.5.3. GLP-1 Receptor Agonists

GLP-1 analogs (such as liraglutide) used for diabetes have shown neuroprotective properties linked to RAGE modulation. Liraglutide administration in diabetic (db/db) mice has been found to improve their cognitive function and concurrently downregulate RAGE expression in the hippocampus [119]. Diabetic mice typically exhibited elevated RAGE levels and reduced brain GLP-1 receptor (GLP-1R) levels. However, liraglutide reversed these trends. In neuronal cell cultures, exposure to AGEs led to RAGE upregulation and reduced cell viability, whereas co-treatment with liraglutide restored viability and prevented RAGE upregulation. Interestingly, no direct physical interaction between GLP-1R and RAGE was found, suggesting that liraglutide’s effect is indirect. It likely acts via improving insulin signaling, reducing AGEs, and activating neurotrophic pathways that counter RAGE’s inflammatory signaling [119]. Clinically, GLP-1 agonists are being explored for cognitive benefits in diabetes and early AD. Reduced RAGE and inflammation in the brain may be one mechanism underlying the memory improvement observed in treated diabetic mice [119]. Thus, incretin-based therapies may confer CNS protection in diabetics by reducing RAGE ligand production (through improved glycemic control) and by activating cell-survival pathways that counteract RAGE’s effects.

#### 4.5.4. Antioxidants

Chronic hyperglycemia induces oxidative stress, accelerating AGE formation and RAGE activation. Antioxidants, including alpha-lipoic acid (ALA), are known to have antioxidant effects in diabetic neuropathy. A study of 54 patients with diabetes with neuropathy found that oral ALA therapy significantly reduced plasma AGE levels compared with baseline [125]. Although sRAGE levels did not change, the AGE/sRAGE ratio tended to improve, and patients showed reduced oxidative damage markers [125]. ALA’s known benefits in diabetic neuropathy may stem partly from lowering circulating AGEs. By reducing the ligand burden, ALA potentially lessens RAGE overstimulation in vessels and nerves, thereby improving endothelial function and nerve blood flow [125]. Other antioxidants (N-acetylcysteine, vitamin E, polyphenols) have likewise been reported in preclinical studies to reduce AGEs or block RAGE’s oxidative signaling, ultimately protecting neurons from glucotoxic stress. For instance, flavonoids like quercetin can inhibit the formation of AGEs and have been noted to decrease RAGE expression in diabetic animal models [144]. In summary, while these agents do not target RAGE directly, they can mitigate upstream or downstream components of the RAGE axis by reducing AGE production, scavenging ROS, or interrupting RAGE-activated inflammation, thereby exerting neuroprotective effects in diabetic conditions (Table 2).

### 4.6. Potential Novel Therapeutics Indirectly Targeting RAGE-Ligand Binding—Alagebrium (ALT-711) and C-Phycocyanin

Alagebrium was the first drug candidate to be clinically tested as an AGE inhibitor, thanks to its ability to break AGE cross-links, tested and tried in phase III clinical trials for cardiovascular disease [145]; however, the company did not decide to put it on the market. Recently, however, the search for a novel treatment of diabetic wound-healing brought a renewed interest in Alagebrium, making it a promising candidate for diabetes-related foot ulcers and wounds [146] (Table 3).

C-phycocyanin is an algae-derived pigment–protein complex and through its mechanism of action reduces production of AGEs and ROS, making it a potential drug candidate in the treatment of type 2 diabetes and its complications [147,148]. C-phycocyanin belongs to the category of biotherapeutics, obtained by extraction from cyanobacteria, mainly Spirulina platensis and Plectonema species [132] and has proven to be effective in attenuating neurodegenerative changes driven by long-term hyperglycemia [132] (Table 3).

**Table 3:** Potential novel therapeutics targeting indirectly RAGE signaling and attenuating diabetes triggered complications.

Drug/Agent	Short Characteristics	Mechanism of Action	Development Stage	Key Reference(s)
C-phycocyanin (C-PC)	Algae-derived pigment–protein complex; likely BBB permeable	Strong antioxidant and antiglycation activity; suppresses RAGE-driven endoplasmic reticulum stress and mitochondrial apoptosis	Preclinical (cell cultures, mouse models); biotherepeutic candidate for diabetes-associated neurodegeneration	[132,147,149,150]
Alagebrium (ALT-711)	Thiazolium derivative; no clear data on BBB permeability	Cleaves pre-formed AGE cross-links in extracellular matrix, reducing tissue stiffness and AGE load	Preclinical (rodent and cell culture) and phase III clinical trials;	[145,146,151]

## 5. Unresolved Questions, Limitations and Conflicting Findings

Despite progress in unrevealing the role of RAGE signaling pathways in neurodegeneration in diabetes, there are still unsolved questions and limitations, slowing down the process of obtaining a wider view of the role of RAGE axis in hyperglycemia-driven neurodegeneration. Most studies use rodents with pharmacologically induced, insulin-dependent type 1 diabetes, while in human population most patients have non-insulin dependent type 2 diabetes making the comparisons less reliable. Furthermore, disease duration differs, and while in animal models, diabetes lasts weeks or months, patients live with diabetes for years or decades before any clinical signs of CNS neurodegeneration are noticeable. The shorted disease duration may favor more acute oxidative or inflammatory cascades over slower neurophysiological adaptations present in patients with diabetes, skewing the view on the pathogenesis of the disease. Furthermore, rodent RAGE signaling, microglial phenotypes, and BBB properties differ from humans, limiting direct extrapolation of neurodegenerative mechanisms and treatment effects. Another limitation is the scarcity of pediatric/adolescent studies on RAGE/AGE biomarkers making it hard to extrapolate available evidence in order to find novel, more efficient and safer treatment of consequences of long-term diabetes.

There are also unresolved questions about therapeutic and biomarker potential of RAGE-ligand signaling partners. Available RAGE inhibitors (e.g., FPS ZM1) may have off target effects, limited CNS penetration, or non-physiological dosing schedules. In addition, many studies lack full pharmacodynamic characterization, making it unclear whether negative or positive results reflect target engagement. AGE and sRAGE levels in blood or CSF are inconsistently measured, and longitudinal human data linking these markers to imaging or cognitive decline in diabetes are still sparse. This limits validation of preclinical findings and hinders patient stratification for RAGE targeted therapies [152,153,154].

Finally, there is some conflicting data on the role of RAGE in nervous system and while most evidence points out to its detrimental effects, some data shows that RAGE and its ligands in low concentrations might provide some neuronal protection. These dual roles of the RAGE-ligand axis have been detailed in one of our earlier study [155]. The summarizing table listing main limitations given below (Table 4).

## 6. Conclusions and Future Directions

In conclusion, despite the limitations and unresolved issues mentioned above, the expanding body of original research supports the notion of the RAGE axis as a central instigator of neurodegeneration in the diabetic brain and spinal cord. New studies have validated prior mechanisms (RAGE-NF-κB inflammation, AGE accumulation) with rigorous in vivo evidence [117,118], while also unveiling novel molecular interactions (such as RAGE-RIPK1 in microglia [117]) that drive neuroinflammatory damage. Clinical investigations confirm these findings, associating RAGE pathway biomarkers with cognitive decline in diabetes [120]. Large-animal models also replicate the RAGE mediated neuropathology observed in rodents [121]. A range of pharmacological interventions, including diabetes medications (metformin, pioglitazone, liraglutide) and antioxidants, has shown efficacy in attenuating diabetes-driven neurodegenerative changes in CNS. It happens at least partly by modulating the RAGE axis or its ligands [119,125,139,141]. A couple of promising therapeutic agents such as small molecule RAGE inhibitors or C-phycocyanin have been tried, tested or are being re-purposed (Alagebrium) for diabetes-related neurodegeneration.

Future studies should focus on dissecting the role of RAGE in relation to different cell types, i.e., glial, neuronal and endothelial cells and regions in CNS, by using RAGE conditional knockouts together with assorted cell lines. Furthermore, future research should prioritize a clear demarcation between RAGE-dependent and RAGE-independent effects in diabetes-driven neurodegenerative changes, making it easier to design on-target treatment.

## Figures and Tables

**Table 1:** Direct RAGE antagonists—short characteristics and mechanisms of action.

Drug/Agent	Short Characteristics	Mechanism of Action	Development Stage	Key Reference(s)
Azeliragon (TTP488)	Orally bioavailable small-molecule RAGE inhibitor; BBB permeable	Binds RAGE and blocks extracellular ligand (AGE, HMGB1, S100) interactions, inhibiting downstream RAGE signaling pathways	Phase II/III trials in AD; not yet approved, but mechanistic template for RAGE blockade in diabetic neurodegeneration	[128,129,130]
FPS-ZM1	High-affinity RAGE inhibitor; BBB permeable	Blocks amyloid β binding and inhibits RAGE-mediated ROS, and inflammatory signaling in vitro (cell cultures) and in vivo (mouse brain)	Preclinical (mouse and cell culture studies); tool compound for proof-of-concept in RAGE-driven neurotoxicity studies	[131,132,133,134]
sRAGE (soluble, endogenous secretory <es> RAGE)	Circulating extracellular domain of RAGE; BBB permeable	Sequesters AGEs and other ligands, preventing their binding to membranous RAGE.	Preclinically used in mouse model of ALS and in human brain endothelial cell	[135,136,137,138]

**Table 2:** Indirect modulation of RAGE in CNS neurodegeneration in diabetes—summary.

Agent/Class	Mechanism of Action	Neuroprotective Effects/Findings
Metformin	- Improves glycemic control	- Decreased RAGE expression in brain vasculature
	- Reduces AGE/RAGE-mediated damage	- Reduced Aβ influx across BBB
	- Downregulates RAGE via AMPK	- Improved memory in diabetic mice
		- Slowed brain aging in macaques
		- Lower markers of inflammation and metabolic stress
Thiazolidinediones (e.g., Pioglitazone)	- PPAR-γ agonist	- Reversed learning/memory impairments in diabetic mice
	- Suppresses RAGE, NF-κB, BACE1	- Lowered RAGE and cytokines in hippocampus
	- Reduces AGE production	- Improved cognition and maze performance
	- Transcriptional repression of RAGE axis	- Reduced IL1β and TNFα in brain
		- Improved memory via anti-inflammatory effects
GLP-1 Receptor Agonists (e.g., Liraglutide)	- Indirectly downregulates RAGE	- Improved cognitive function in diabetic mice
	- Improves insulin signaling	- Downregulated RAGE in hippocampus
	- Reduces AGEs	- Restored neuronal viability
	- Activates neurotrophic pathways	- Reduced RAGE elevation
		- Potential cognitive benefits in diabetes and early AD
Antioxidants (e.g., Alpha-lipoic acid, N-acetylcysteine, Vitamin E, Polyphenols)	- Reduce oxidative stress	- Decreased plasma AGE concentrations
	- Lower AGE formation	- Improved AGE/sRAGE ratio
	- Block RAGE oxidative signaling	- Reduced oxidative damage markers
		- Improved endothelial function and nerve blood flow
		- Flavonoids (e.g., quercetin) inhibit AGE formation and decrease RAGE expression

**Table 4:** Main limitations of current models used in studies of RAGE axis in CNS neurodegeneration in long-term hyperglycemia.

Key Issue	Main Limitation	Relevance
Disease duration	Short duration—weeks or months in animal models vs. years or decades in patients with diabetes	Impaired translation of results from animal studies to clinical trials
Species/strain	Rodent-restricted, few strains	Limited generalizability and immune/metabolic mismatch
RAGE biology	Oversimplified view of “RAGE = bad”	Missed ligand diversity, co-receptors, and compensatory pathways
CNS specificity	Most studies performed on peripheral nervous system; brain and spinal cord less studies	Understudied relation between RAGE signaling and defined neural circuits
Clinical application	Sparse human longitudinal data; lack of data from pediatric/adolescent patients	Hard to validate targets, biomarkers, and therapies

## Data Availability

No new data were created or analyzed in this study. Data sharing is not applicable to this article.

## Abbreviations

The following abbreviations are used in this manuscript:

RAGE	Receptor for advanced glycation end-products
AGEs	Advanced glycation end-products
ROS	Reactive oxygen species
HMGB1	High mobility group box 1
TLR	Toll-like receptor
BBB	Brain–blood barrier
SMA	Spinal muscular atrophy
CNS	Central nervous system
Pfn	Profilin
TNF	Tumor necrosis factor
ROCK	RhoA-associated kinase
MGO	Methylglyoxal
AD	Alzheimer’s disease
SMN	Survival motor neuron
CML	N-epsilon-carboxymethyl lysine
DRF	Diaphanous-related formins
IL	Interleukin
GO	Glyoxal
RIPK1	Receptor-Interacting Protein Kinase
NLRP3	Nucleotide-binding and oligomerization (NOD) like receptor (NLR)
BDNF	Brain-derived neurotrophic factor
ALS	Amyotrophic lateral sclerosis
Aβ	Amyloid beta
ICAM	Intercellular adhesion molecule
JNK	c-Jun N-terminal kinase
MAPK	Mitogen-activated protein kinase
NF-κB	Nuclear factor-kappa B
